# Self-reported anosmia and dysgeusia as key symptoms of coronavirus disease 2019

**DOI:** 10.1017/cem.2020.420

**Published:** 2020-06-08

**Authors:** Daniel J. Lee, Joel Lockwood, Paul Das, Ri Wang, Eitan Grinspun, John M. Lee

**Affiliations:** * Department of Otolaryngology – Head and Neck Surgery, St. Michael's Hospital, Unity Health Toronto, University of Toronto, Toronto, ON; † Li Ka-Shing Knowledge Institute, St. Michael's Hospital, Unity Health Toronto, Toronto, ON; ‡ Department of Emergency Medicine, St. Michael's Hospital, Unity Health Toronto, Toronto, ON; § Department of Family and Community Medicine, St. Michael's Hospital, Unity Health Toronto, Toronto, ON; ¶ MAP Centre for Urban Health Solutions, Li Ka-Shing Knowledge Institute, St. Michael's Hospital, Unity Health, Toronto, ON; ** Department of Computer Science, University of Toronto, Toronto, ON

**Keywords:** Anosmia, COVID-19, dysgeusia, epidemiology, population health

## Abstract

**Objectives:**

To slow down the transmission of coronavirus disease 2019 (COVID-19), it is important to identify specific symptoms for effective screening. While anosmia/hyposmia and dysgeusia/ageusia have been identified as highly prevalent symptoms, there are wide geographic variations, necessitating the regional evaluation of the prevalence of the symptoms.

**Methods:**

A cross-sectional study was performed to evaluate the self-reported symptoms among adults (over 18 years old) who underwent COVID-19 tests at an ambulatory assessment centre. We identified 1,345 patients (102 positive and 1,243 negative) who visited the assessment centre between March 16 and April 15, 2020. We randomly sampled negative patients in a 1:3 ratio. The primary outcome was the prevalence of self-reported anosmia/hyposmia and dysgeusia/ageusia. Logistic regression was performed to evaluate the association between COVID-19 positivity and loss of smell and taste.

**Results:**

Fifty-six of 102 (50%) positive patients and 72 of 306 (23.5%) negative patients completed the survey. Anosmia/hyposmia and dysgeusia/ageusia were more prevalent among COVID-19 positive patients (41.1% v. 4.2%, *p* < 0.001 for smell and 46.4% v. 5.6%, *p* < 0.001 for taste). Anosmia/hyposmia and dysgeusia/ageusia were independently highly associated with COVID-19 positivity (adjusted odds ratios 14.4 and 11.4 for smell and taste, respectively).

**Conclusion:**

In this Canadian study, smell and taste loss may be key symptoms of COVID-19. This evidence can be helpful in the clinical diagnosis of COVID-19, particularly settings of limited testing capacity.

## RÉSUMÉ

**Contexte:**

Afin de ralentir la transmission de la maladie à coronavirus 2019 (COVID-19) par un dépistage efficace, il est important de reconnaître les symptômes spécifiques de la maladie. Bien que l'anosmie/l'hyposmie et la dysgeusie/l'ageusie soient souvent des symptômes concomitants de la maladie, il existe de grandes variations géographiques qui appellent une évaluation régionale de la prévalence des symptômes.

**Méthode:**

Il s'agit d'une étude transversale visant à évaluer la pertinence de symptômes autodéclarés de la maladie chez des adultes (plus de 18 ans) ayant subi un test de dépistage de la COVID-19 dans un centre ambulatoire de prélèvements. Ainsi, 1345 patients (102 cas positifs et 1243 cas négatifs) se sont rendus dans un centre de dépistage entre le 16 mars et le 15 avril 2020. L’équipe a par la suite procédé à un échantillonnage aléatoire de patients ayant obtenu un résultat négatif dans un rapport de 1/3. Le critère d’évaluation principal était la prévalence des symptômes autodéclarés d'anosmie/d'hyposmie et de dysgeusie/d'ageusie. L'association entre la positivité des résultats à l’égard de la COVID-19 et la perte de l'odorat et du goût a été examinée à l'aide d'une régression logistique.

**Résultats:**

Dans l'ensemble, 56 patients sur 102 (50%) déclarés positifs et 72 patients sur 306 (23,5%) déclarés négatifs ont poursuivi l'enquête jusqu'au bout. La prévalence de l'anosmie/l'hyposmie et de la dysgeusie/l'ageusie était plus élevée chez les patients déclarés positifs (odorat : 41,1% contre [c.] 4,2%, *p* < 0,001; goût : 46,4% c. 5,6%, *p* < 0,001) que chez ceux déclarés négatifs. L'anosmie/l'hyposmie et la dysgeusie/l'ageusie se sont révélées des symptômes indépendants, fortement associés à la positivité des résultats à l’égard de la COVID-19 (risque relatif approché rajusté : 14,4 et 11,4 pour l'odorat et le goût, respectivement).

**Interprétation:**

Compte tenu des résultats obtenus dans cette étude réalisée au Canada, la perte de l'odorat et du goût pourrait être considérée comme un symptôme important de la maladie. Il s'agirait là d'une manifestation susceptible de faciliter la pose du diagnostic clinique de la COVID-19, tout particulièrement dans les milieux où la capacité de dépistage est restreinte.

## CLINICIAN'S CAPSULE

**What is known about the topic?**

Patients with coronavirus disease 2019 (COVID-19) present with various influenza-like symptoms, making it difficult to distinguish from other viral infections for frontline physicians.

**What did this study ask?**

Do COVID-19 positive patients have higher prevalence of self-reported anosmia/hyposmia and dysgeusia/ageusia than COVID-19 negative patients?

**What did this study find?**

This study found that anosmia/hyposmia and dysgeusia/ageusia were respectively 14 and 11 times more likely to be associated with COVID-19 positivity.

**Why does this study matter to clinicians?**

Using anosmia/hyposmia and dysgeusia/ageusia as specific criteria may lead to improved clinical diagnosis of COVID-19 positive patients.

## INTRODUCTION

Coronavirus disease 2019 (COVID-19) is an international outbreak of respiratory illness characterized by high transmission rate, particularly in individuals with no or mild symptoms.^1,2^ A key step in minimizing transmission is to screen and isolate those infected with COVID-19 using specific clinical criteria. While several symptoms are used to screen, there is lack of data to suggest specific symptoms of COVID-19. Anosmia/hyposmia and ageusia/dysgeusia have emerged as potential specific symptoms, as reported in recent studies from the United States and Europe.^3–5^ Spinato et al. described 64.4% of altered smell or taste among Italian adults with mild symptoms and Yan et al. described 68% and 71% of anosmia and dysgeusia, respectively, in COVID-19 positive individuals with an odds ratio (OR) of 10.^3,4^ A recently published study from Quebec reported 51.5% of anosmia.^6^ These reports suggest that anosmia/hyposmia and dysgeusia/anosmia have the potential to be used to specifically screen for individuals with mild symptoms. However, there are wide variations in the reported prevalence of anosmia/hyposmia and ageusia/dysgeusia across different regions, potentially suggesting variable geographic presentations of severe acute respiratory syndrome coronavirus 2 (SARS-CoV-2).^7^ Therefore, it is imperative that we further examine this potential association between loss of smell and taste and COVID-19 diagnosis in the Canadian and Ontario context. The objective was to evaluate the prevalence and association of anosmia/hyposmia and dysgeusia/ageusia in patients who underwent COVID-19 testing using self-reported measures.

## METHODS

### Study design and patient population

The approval of this study was granted by the Research Ethics Board at Unity Health Toronto, Toronto, Ontario, through Clinical Trials Ontario (CTO ID: 2142). We designed a cross-sectional survey of adults (over 18 years of age) who had undergone polymerase chain reaction–confirmed COVID-19 testing via nasopharyngeal swab at the COVID-19 Assessment Centre at St. Michael's Hospital, Toronto, Ontario, between March 16 and April 15, 2020. Patients were contacted by phone for an invitation to a secure online survey, which was constructed using Snap Software, fully compliant with the Personal Health Information Protection Act, after being notified of the results of their swab. During this period, we identified 102 unique COVID-19 positive patients and 1,243 unique negative patients. Due to a large number of negative patients, we randomly sampled negative patients in a 1:3 ratio.

### Outcome measures and data collection

Baseline characteristics were collected and included: age, gender, medical comorbidities, and smoking status. We listed chronic rhinosinusitis and history of recent severe upper respiratory tract infection (URTI) or flu as a separate comorbidity, as these may impact baseline sense of smell. COVID-19 diagnosis, symptoms, and hospitalizations were collected. Smell and taste-specific questions included the presence of smell or taste loss around the onset of COVID-19-like symptoms (5 days earlier or any time after), as well as the current ability to smell. The type of taste loss was collected (sweet, salty, sour, bitter, and savoury).

### Statistical analysis

Demographic and clinical characteristics were summarized descriptively by reporting the median and interquartile (IQR) range for continuous variables and the frequency and proportion for categorical variables. Differences in characteristics between two comparison groups were compared using the Kruskal-Wallis test for continuous variables and Fisher's exact test for categorical variables. Unadjusted ORs were calculated with univariable logistic regression. Adjusted OR was calculated using multivariable logistic regression analysis with a priori criteria. To prevent over-fitting, we included the loss of smell or taste around the onset of COVID-19-like symptoms and four other covariates with a statistically significant magnitude of association of at least twofold (i.e., OR > 2.0 or OR < 0.5).

## RESULTS

Of 102 positive patients and 306 negative patients who were contacted, 56 (50%) positive patients and 72 (23.5%) negative patients completed the survey between April 15 and May 1, 2020. Baseline information is summarized in Table 1. Positive patients were younger than negative patients (38.0 IQR 31.8–47.2 v. 43.0 IQR 33.5–55.0, *p* < 0.05). Gender, smoking status, and comorbidities relevant to smell function (head trauma, chronic rhinosinusitis, and recent URTI/flu) were well-balanced. There was a longer time lapse between the diagnosis and the survey among the negative patients compared with the positive patients (67.6% for negative v. 30.4% for positive for more than 4 weeks since the diagnosis).

**Table 1.** Comparisons of baseline characteristics of groups by COVID-19 diagnosis

	Total (n = 127)	Positive (n = 56)	Negative (n = 71)
Age – median (IQR)*	41.0 (32.0–52.5)	38.0 (31.8–47.2)	43.0 (33.5–55.0)
Gender – %			
Male	38.6	41.1	36.6
Female	60.6	58.9	62.0
Others	0.8	0.0	1.4
Smoking – %			
Never	67.7	71.4	64.8
Current	9.5	5.4	12.7
Ex-smoker	21.3	19.6	22.5
Others (vape)	1.6	3.6	0.0
Comorbidities – %			
Cardiac	1.6	0.0	2.8
Neurological	0.0	0.0	0.0
Respiratory	14.2	17.9	11.3
Diabetes	7.1	7.1	7.0
Hypertension	13.4	10.7	15.5
Cancer*	5.5	0.0	9.9
Head trauma	5.5	3.6	7.0
Chronic rhinosinusitis	3.9	3.6	4.2
Recent severe URTI or flu	9.5	8.9	9.9
None*	38.6	25.0	49.3
Time of survey completion from time of swab – %			
0–2 weeks	14.2	30.4	1.4
2–4 weeks	34.7	39.3	31.0
>4 weeks	51.2	30.4	67.6
Hospitalization – n (%)	7.0	7.3	0.0
Overall symptoms – n (%)			
Sore throat*	52.0	37.5	63.4
Cough*	52.8	66.1	42.3
Nasal congestion	39.4	41.1	38.0
Rhinorrhea	36.2	26.8	43.7
Fever*	35.4	46.4	26.7
Shortness of breath	31.5	37.5	26.8
Abdominal pain	10.2	12.50	8.5
Diarrhea*	26.0	35.7	18.3
Anosmia*	20.5	42.9	2.8
Hyposmia*	6.3	12.5	1.4
Dysgeusia/ageusia*	26.0	57.1	1.4
Fatigue	11.8	7.1	15.5
Headache*	11.0	17.9	5.6
Other**	35.4	44.6	28.2

Notes:

*Statistically significant *p*-value (< 0.05). Kruskal-Wallis test for continuous variables and Fisher's exact test for categorical variables.

**Includes nonspecific symptoms, such as body aches, dizziness, ear pain, and myalgia with small prevalence.IQR = interquartile range; URTI = upper respiratory tract infection.

The overall symptoms are summarized in Table 1. A significantly higher proportion of positive patients reported anosmia (42.9% v. 2.8%, *p* < 0.001), hyposmia (12.5% v. 1.4%, *p* < 0.05) and dysgeusia/ageusia (57.1% v. 1.4%, *p* < 0.001) compared with negative patients. In addition, cough, fever, diarrhea, and headache were more common among positive patients. On the other hand, sore throat was more common among negative patients (63% v. 37%, *p* < 0.01).

Characterization of anosmia/hyposmia and dysgeusia/ageusia is summarized in Table 2. When patients were inquired about their smell loss around the time of COVID-19-like symptom onset (5 days before or any time after), there was a significantly higher proportion of COVID-19-positive patients compared with the negative patients (41.1% v. 4.2%, *p* < 0.001). Of 23 positive patients with anosmia/hyposmia, 12 (52.2%) patients reported that anosmia/hyposmia was one of the early symptoms. There was a significantly higher proportion of positive patients reporting dysgeusia/ageusia compared with negative patients (46.4% v. 5.6%, *p* < 0.001). Twenty (35.7%) positive patients reported concomitant smell and taste loss.

**Table 2.** Characterization of anosmia/hyposmia and ageusia/dysgeusia by COVID-19 diagnosis

Characterization of anosmia/hyposmia – %			
	Total (n = 127)	Positive (n = 56)	Negative (n = 71)
Anosmia/hyposmia around the time of COVID-19 symptom onset* (5 days before or any time after)			
Yes	20.5	41.1	4.2
No	64.6	41.1	83.1
Unable to recall	13.4	16.1	11.3
Prefer not to respond	0.8	0.0	1.4
Timing of anosmia/hyposmia	Total (n = 26)	Positive (n = 23)	Negative (n = 3)
Before any other symptoms	11.5	8.7	33.3
Early (after one or two other symptoms)	42.3	43.5	33.3
Late (after other symptoms developed)	42.3	43.5	33.3
Unknown (did not select)	3.8	4.3	0.0
Current ability to smell	Total (n = 26)	Positive (n = 23)	Negative (n = 3)
Normal	50.0	47.8	66.7
Diminished	23.1	26.1	0.0
Absent	23.1	26.1	0.0
Unknown (did not select)	3.9	0.0	33.3
Characterization of dysgeusia/ageusia – %			
	Total (n = 127)	Positive (n = 56)	Negative (n = 71)
Dysgeusia/ageusia around the time of COVID-19 symptom onset* (5 days before or any time after)			
Yes	23.6	46.4	5.6
No	69.3	51.8	83.1
Unable to recall	6.3	1.8	9.9
Prefer not to respond	0.8	0.0	1.4
Component of taste loss	Total (n = 30)	Positive (n = 26)	Negative (n = 4)
Salty	73.3	73.1	75.0
Sweet	60.0	65.4	25.0
Sour	56.7	57.7	50.0
Bitter	60.0	61.5	50.0
Savoury	76.7	80.8	50.0

*Statistically significant *p*-value (< 0.05). Kruskal-Wallis test for continuous variables and Fisher's exact test for categorical variables.

On the univariable analysis, anosmia/hyposmia and dysgeusia/ageusia were highly associated with COVID-19 positivity OR 19.7 (95% confidence interval [CI] 6.1–88.7) and OR 13.2 (95% CI 4.6–48.0), respectively (Table 3). Fever, cough, sore throat, and headache were included in the multivariable analysis with either anosmia/hyposmia or dysgeusia/ageusia. In the multivariable models, smell loss and taste loss demonstrated high adjusted ORs with COVID-19 positivity (OR 14.4 [95% CI 4.0–70.5] and OR 11.4 [95% CI 3.6–45.3], respectively). Sore throat was negatively associated with COVID-19 positivity. Other symptoms were statistically not significant.

**Table 3.** Univariable and multivariable analysis for the association between influenza-like symptoms and COVID-19 diagnosis

	Univariable analysis		Multivariable analysis with anosmia/hyposmia		Multivariable analysis with dysgeusia/ageusia	
	OR (95% CI)	*p*-value	OR (95% CI)	*p*-value	OR (95% CI)	*p*-value
Age	1.0 (0.9–1.0)	**0**.**04**				
Gender	1.2 (0.6–2.4)	0.7				
Fever	2.4 (1.1–5.1)	**0**.**02**	1.9 (0.7–5.3)	0.2	1.8 (0.7–4.7)	0.3
Cough	2.7 (1.3–5.6)	**< 0.01**	1.8 (0.7–5.2)	0.3	2.2 (0.9–5.8)	0.1
Sore throat	0.4 (0.2–0.7)	**< 0.01**	0.3 (0.1–0.8)	**0**.**01**	0.3 (0.09–0.6)	**< 0.01**
Rhinorrhea	0.5 (0.2–1.0)	**0**.**05**				
Nasal congestion	1.1 (0.6–2.3)	0.7				
Shortness of breath	1.6 (0.8–3.5)	0.2				
Fatigue	0.4 (0.1–1.3)	0.2				
Headache	3.6 (1.1–13.9)	**0**.**04**	3.6 (0.7–22.0)	0.1	3.7 (0.8–20.6)	0.1
Abdominal pain or diarrhea	2.2 (1.0–5.0)	**0**.**04**				
Anosmia/hyposmia*	19.7 (6.1–88.7)	**< 0.001**	14.4 (4.0–70.5)	**< 0.001**		
Dysgeusia/ageusia*	13.2 (4.6–48.0)	**< 0.001**			11.4 (3.6–45.3)	**< 0.001**

*Notes:* Boldface text indicates statistically significant *p*-value (< 0.05).

*Smell and taste loss based on reported data around the time of self-reported COVID-19 symptom onset (5 days before or any time after); OR = odds ratio; CI = confidence interval.

There were no differences between patients with and without anosmia/hyposmia in terms of age, smoking, relevant comorbidities (chronic rhinosinusitis, recent URTI/flu, head trauma), and symptoms (rhinorrhea and nasal congestion) (Table 4). There were significantly more COVID-19-positive patients in the group with smell loss than the one without (88.5% v. 28.1%, *p* < 0.001).

**Table 4.** Comparison of clinically relevant variables between the group with and without anosmia/hyposmia

	Total (n = 108)	Anosmia/hyposmia (n = 26)	No anosmia/hyposmia (n = 82)
Age – median (IQR)	40.0 (32.0–52.3)	36.0 (31.0–45.3)	42.0 (32.3–53.0)
Smoking status – %			
Current	7.4	3.9	8.5
Ex-smoker	20.4	15.4	22.0
Never	70.4	76.9	68.3
E-cigarettes	1.9	3.9	1.2
Relevant comorbidities – %			
Chronic rhinosinusitis	4.6	0.0	6.1
Recent severe URTI or flu	11.1	15.4	9.8
Head trauma	6.5	0.0	8.5
Relevant symptoms –%			
Rhinorrhea	37.0	23.1	41.5
Nasal congestion	40.7	46.2	39.0
COVID-19 status* – %			
Positive	42.6	88.5	28.1
Negative	57.4	11.5	72.0

Notes:

*Statistically significant *p*-value (< 0.05). Kruskal-Wallis test for continuous variables and Fisher's exact test for categorical variables.

IQR = interquartile range; URTI = upper respiratory tract infection.

Among those with anosmia/hyposmia (n = 25, 1 did not answer), 13 (52%) patients reported their sense of smell at the time of survey to be normal. Twelve (48%) patients who had persistent smell loss were within less than 4 weeks from the diagnosis. All eight patients who completed the survey more than 4 weeks after the diagnosis reported a normal sense of smell.

## DISCUSSION

### Interpretation of findings

This study suggests that there is a higher prevalence of self-reported chemosensory impairment in COVID-19-positive patients compared with negative patients with early recovery of smell function in a large proportion of patients. In our series, olfactory and gustatory impairments were respectively 14 times and 11 times more likely to be associated with COVID-19 positivity. Overall, our findings suggest that loss of smell and loss of taste have a higher chance of identifying COVID-19-positive patients among those with influenza-like symptoms seen on an ambulatory basis.

The reported prevalence of olfactory dysfunction varies significantly from 5.1% in a Chinese study to 85.6% in a European study.^3–5,7–9^ Our results are consistent with previous reports that showed a higher prevalence of self-reported smell or taste loss among COVID-19-positive patients. Interestingly, in our study, although 31 out of 56 (55.4%) of COVID-19-positive survey participants included anosmia or hyposmia as one of their overall symptoms, only 23 (41.1%) patients maintained that they lost their sense of smell around the time of COVID-19 symptom onset (5 days prior or thereafter) with a 16% rate of “unable to recall.” This discrepancy likely represents recall bias, which is common in cross-sectional studies. Therefore, we believe that the 40% rate of smell loss around the time of COVID-19 symptom onset is a more accurate representation than the 55% overall rate.

### Comparison with previous studies

Our rate of anosmia/hyposmia of 41.1% is lower than the rate of 60%–85% in recently published studies with comparable study designs or 51.5% found in another Canadian study.^3,4,6,8^ There may be several explanations for this difference. One consideration is the cultural difference in olfactory perception.^10–12^ In fact, participants in other cultures may have different thresholds for their smell loss. Another possible explanation is the presence of mutant strains with varying pathogenicity, as evidenced from genomic studies in multiple nations.^13–16^ These different strains may cause varying degrees of chemosensory impairment with regional discrepancy, although this has not been scientifically proven. Therefore, it is important to collect and examine regional data to depict a representative landscape for our population. Lastly, we do note that this difference could be a simple function of recall bias from cross-sectional surveys, as other studies do not report an “unable to recall” option, thus forcing patients to choose with an altered response rate of self-reported smell loss. Regardless, all published studies unanimously support the inclusion of smell and taste loss as important markers of COVID-19.

### Clinical and research implications

A possible mechanism of olfactory dysfunction caused by SARS-CoV-2 has been demonstrated in experimental models with inoculation of coronaviruses. Coronaviruses can damage the olfactory neuroepithelium via apoptosis with a subsequent reduction of mature sensory neurons and disordering of olfactory epithelium.^17–19^ Another line of research suggests that the anosmia/hyposmia may result from infection of the support and perivascular cells in olfactory epithelium, via angiotensin-converting enzyme 2 receptors, which are identified as the receptors of SARS-CoV-2.^20,21^ Clinically, nasal inoculation of coronavirus resulted in olfactory impairment in healthy volunteers.^22^ Dysgeusia/ageusia is generally regarded to be secondary to loss of smell, as it may be difficult to distinguish between flavor and taste. In a large study investigating taste disorders unrelated to COVID-19, many reported loss of taste without any objective gustatory loss but found to have olfactory deficits alone.^23^ While it is possible that loss of taste in COVID-19 may be a distinct mechanism,^24^ the rate of taste loss in our patients likely represents subtle olfactory deficits. In our series, 52% of the affected individuals reported a normal sense of smell at the time of survey with many recovering within 4 weeks. This finding is consistent with previous reports and suggests that the damage to the olfactory function by COVID-19 may not be permanent.^4^ However, this warrants further investigation with follow-up assessments of smell function. All in all, the presence of loss of smell and taste may be a distinguishable feature of COVID-19 from other viral URTIs.

Our results have strong implications in public health measures. A recent study demonstrated 56% of residents to be COVID-19 positive but “asymptomatic” in a skilled nursing facility.^25^ However, this study did not include the loss of smell or taste in the symptom assessment. In a previous study from Italy and our study, both demonstrate that a significant portion of the patients complained of decreased sense of smell or taste during the early phase of their disease.^3^ Hence, it would be essential to incorporate anosmia/hyposmia and dysgeusia/ageusia as part of future epidemiologic COVID-19 surveillance studies and tailor the screening criteria for COVID-19 accordingly. Furthermore, these symptoms can be easily assessed in the context of telemedicine and virtual care. This can, in turn, facilitate improved patient counselling for the screening of COVID-19 and help curb the spread of COVID-19 in the vulnerable population, including those in long-term care facilities and remote communities where testing may be limited. Currently, while several provincial bodies have implemented loss of smell/taste as screening symptoms, the self-assessment tool from the Canadian Federal Government still does not incorporate these symptoms. With our Ontario regional data demonstrating a high probability of COVID-19 positivity with loss of smell/taste, there is now evidence to expand public awareness of this association and ultimately improve our public health response to this pandemic.

### Limitations

Interpretation of our study results is limited by recall bias, as there is a significant time lapse between diagnosis and survey administration. The recent media spotlight of COVID-19 and anosmia might have resulted in elevated rates of self-reported anosmia. We tried to mitigate this issue by asking similar, but differently worded, questions for internal validity. Future studies could be administered at the time of swab to avoid this bias. We also acknowledge non-response bias. While the study results are limited by the nature of a single-institutional-based study, we collected data over a longer period of time than previous reports to improve our sampling.^3,4^ Furthermore, the testing criteria focused on frontline workers from at-risk settings, such as healthcare or long-term care, residents in group homes, and returning travelers. This may reduce external validity. Lastly, with ethical concerns of transmission to our research team and preservation of personal protective equipment, we are presently unable to objectively measure smell in symptomatic patients during the point of assessment.

We acknowledge the need for large-scale epidemiologic studies to further investigate this association between smell and taste loss and COVID-19 positivity. Our research group currently has ongoing projects to investigate a larger regional population and to incorporate self-reported objective measures of smell using common household items. Despite the limitations, the baseline characteristics between positive and negative groups were comparable, and our data emphasize the importance of considering smell and taste as screening symptoms.

## CONCLUSION

Our results demonstrate that there is a higher rate of self-reported anosmia/hyposmia and concurrent dysgeusia/ageusia among COVID-19 patients in a regional sample of Canadians in Toronto, Ontario. Our study offers strong support for clinicians to use loss of smell and loss of taste as key symptoms for the clinical diagnosis of COVID-19, especially in settings with limited testing. Future population-based studies are needed to further investigate this association.
